# Drying Temperature and Storage Duration for the Retention of Bioactive Compounds of Selected Wild Edible Plants From Ethiopia

**DOI:** 10.1002/fsn3.70782

**Published:** 2025-08-16

**Authors:** Tamene Daba Rumicha, Frafis Hirko, Sirawdink Fikreyesus Forsido, Yetenayet Bekele Tola, Abebe Yimer, Chala G. Kuyu, Tilahun A. Teka, Addisalem Hailu

**Affiliations:** ^1^ Jimma University College of Agriculture and Veterinary Medicine Jimma Ethiopia; ^2^ Faculty of Agriculture, Department of Food and Nutritional Sciences Wollega University Shambu Ethiopia

**Keywords:** β‐carotene, ascorbic acid, bioactive compounds, drying kinetics, flavonoids, phenols, storage duration

## Abstract

Foods rich in bioactive compounds significantly prevent chronic diseases; however, these compounds are susceptible to drying temperature and storage duration because they lose some of their functionalities. This study aimed to determine effective drying temperatures and storage durations for better retention of bioactive compounds of three Ethiopian wild edible plants: *Mussaenda arcuata*, *Celosia trigyna* , and *Pteridium aquilinum* . The collected samples were oven‐dried at 60°C, 65°C, and 70°C in a convective oven dryer to equilibrium moisture content and cooled to room temperature and analyzed for ascorbic acid, β‐carotene, total flavonoid and phenolic contents. To select an appropriate mathematical model for drying, parameters to determine the drying kinetics were also collected simultaneously. The results showed that the dried sample at 60°C exhibited better retention of ascorbic acid, β‐carotene, and flavonoids, except for the phenolic content of *Celosia trigyna* at 65°C. Drying kinetics results in the Henderson and Pabis's model best fitted for *Mussaenda arcuata* and *Celosia trigyna* , while a two‐term model is fitted for *Pteridium aquilinum* . After determining the optimum drying temperature, the samples were then packed with polyethylene bags and stored at room temperature for 12 months. The phytochemical contents were also analyzed at 0, 4, 8, and 12 months to determine the optimal storage time. Storage analysis over 12 months showed significant degradation of bioactive components, particularly ascorbic acid in *Mussaenda arcuata* (47.57% loss within 4 months), with a half‐life time of 4.56 months. Therefore, drying at 60°C and consuming the products in 4 to 6 months ensures better retention of bioactive compounds, supporting the effective use of wild edible plants to promote improved nutrition, sustainable food processing, and improved food and health security in both rural and urban communities.

## Graphical abstract

Wild edible plants are rich in bioactive compounds like ascorbic acid, beta‐carotene, flavonoids, and phenols, which offer significant health benefits but are highly sensitive to heat, light, and oxygen. To preserve these nutrients, drying should be done at optimal temperatures. Proper drying and storage techniques help maintain the nutritional quality and extend the shelf life of these valuable plants.

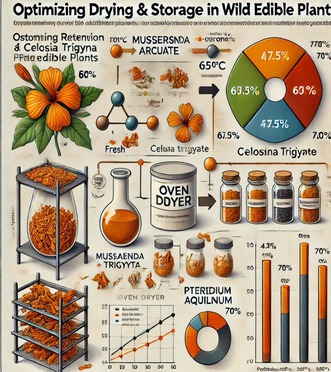

## 1 Introduction

Wild edible plants are considered easily accessible sources of micronutrients, macronutrients, and bioactive compounds for rural communities in Ethiopia, especially during periods of food shortage. Even during favorable rainy seasons, when the fruits ripen, children commonly consume these plants while herding cattle. Many wild edible plants are fruits and vegetables that are potential sources of bioactive compounds such as vitamins (β‐carotene and vitamins C and E) and polyphenols (flavonoids, tannins, and catechins) that have antioxidant activities (Yimer et al. 2023; Jiru et al. 2023; Tsehay et al. 2023; Kwinana‐Mandindi 2015; Deng et al. 2013; Wong et al. 2006). Their great potential makes them good candidates to be produced at the commercial level to contribute to the efforts to secure the food and nutrition of Ethiopia.

*Mussaenda arcata* fruits, *Celosia trigyna* leaves, and *Pteridium aquilinum* fronds are wild edible plants traditionally consumed for both food and medicinal purposes in the southern and southwestern regions of Ethiopia. These species are notable for their high nutritional value, optimal mineral composition, and acceptable levels of antinutritional factors (Rumicha, Belew, et al. 2025; Rumicha, Forsido, et al. 2025).

Bioactive compounds (polyphenols, flavonoids, carotenoids, glucosinolates, alkaloids, and saponins) are naturally present chemical components in plant‐based foods that provide health benefits beyond basic nutrition. These substances exhibit a range of physiological properties in our body. In addition to their nutritional value, antioxidant, anti‐inflammatory, antimicrobial, and cardioprotective activities contribute to the prevention of disease and overall health improvement (Guaadaoui et al. 2014; Kris‐Etherton et al. 2002). They contribute to disease prevention through various interconnected mechanisms, regulation of gene expression, modulation of enzymatic activities, immune system support, and antimicrobial action (Liu et al. 2005; Liu 2004).

However, they are perishable by nature due to their high moisture and nutrient content, which hinders their storage and use during off‐seasons. It is also difficult to harvest and supply to distant markets due to the absence of cold‐chain facilities. However, optimal drying and storage of dried products can help to harvest them during the rainy season and be used or shipped to distant markets.

By removing moisture, drying prevents microbial growth, allowing produce to be stored for months or even years without refrigeration. This method is particularly beneficial in reducing food spoilage, which contributes to the reduction of global food loss (FAO 2019). The convenience of dried foods also makes them ideal for traveling and cooking. Furthermore, drying is an environmentally friendly preservation method that requires less energy compared to freezing or canning (Rahman et al. 2007).

Even with careful harvesting, handling, and processing, bioactive compounds such as vitamin C, polyphenols, flavonoids, carotenoids, and other phytochemicals are highly prone to degradation due to factors like heat, oxidation, and prolonged storage. Among these, vitamin C is particularly sensitive to oxidation and temperature, making it especially vulnerable—even under ideal storage conditions (Klimczak et al. 2007; Vidinamo et al. 2021).

Drying is a key post‐harvest preservation technique for maintaining the shelf life of wild edible plants (WEPs). However, the main challenge in drying biological materials is the loss of heat‐sensitive bioactive compounds. Studies have shown that prolonged exposure to elevated temperatures during drying can significantly reduce the content of vitamin C, phenolics, carotenoids, and flavonoids (Elgamal et al. 2023; Giannakourou and Taoukis 2021). In addition, these compounds are unstable during storage and degrade at varying rates. Over time, cumulative losses reduce the nutritional value and health‐promoting properties of the dried products (Andersson et al. 2015; Debenedictis et al. 2023).

In areas where wild edible plants are abundant, local communities typically harvest, process, and consume them on the same day. Despite this traditional practice, there is limited scientific understanding of how drying methods and storage durations affect the retention of bioactive compounds in WEPs, especially under ambient conditions common in rural Ethiopia. Although traditional drying methods are widely used and help reduce spoilage, very few studies have investigated how different drying techniques impact the bioactive compounds and storage stability of WEPs. This gap in research limits the ability to recommend appropriate post‐harvest practices that preserve the full nutritional and bioactive compound potential of these plants. Furthermore, the drying behavior and kinetics of the samples were investigated, and alternative drying kinetic models were assessed to select and recommend the suitable model for future use.

## 2 Materials and Methods

### 2.1 Description of the Study Sites

**FIGURE 1:**
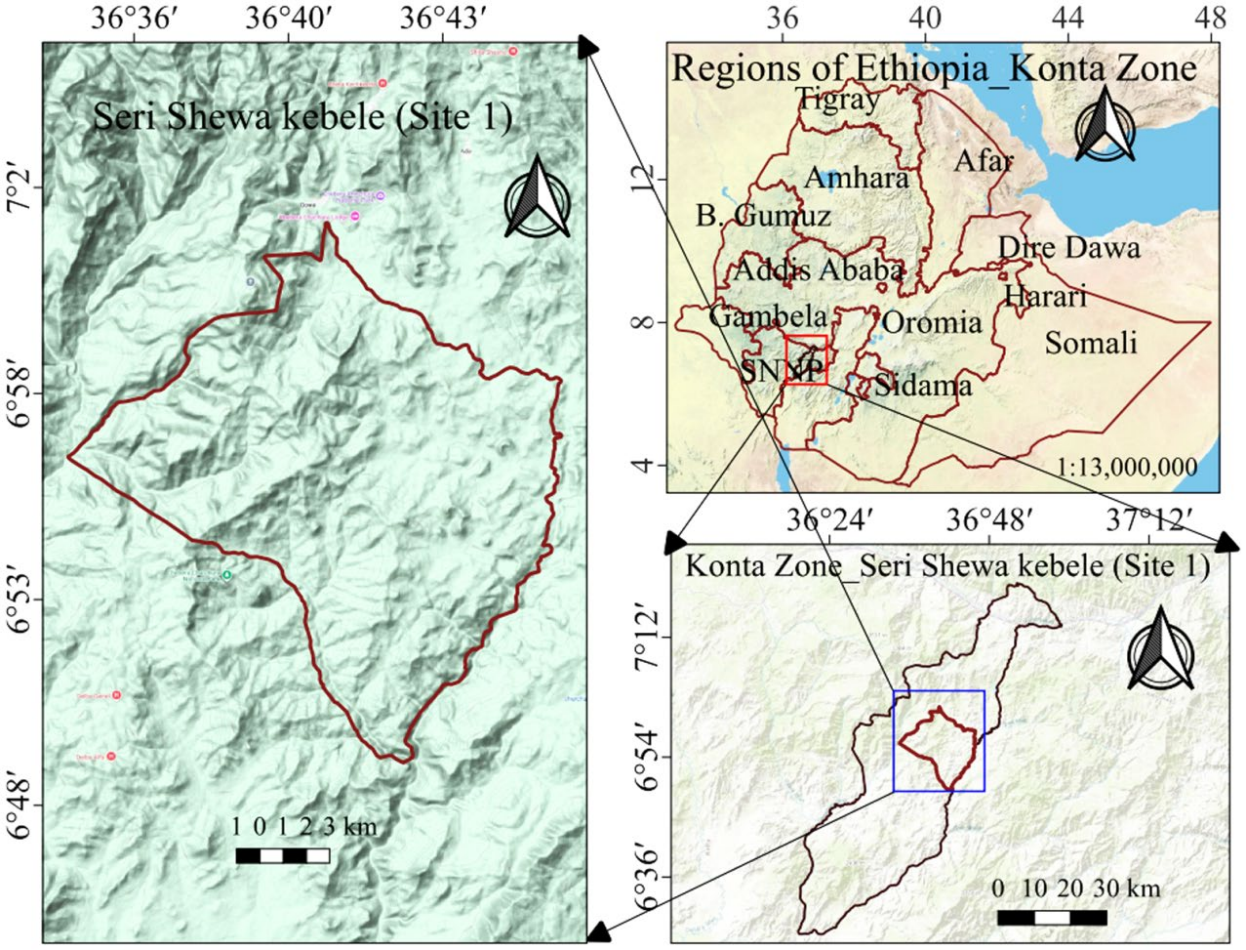
Map of the study site 1 (Seri Shewa Kebele) to collect *Mussaenda arcuata fruits* (locally known as Mentserko).

The study samples were collected from two distinct locations in the southern and southwestern regions of Ethiopia (Figure 1 and 3). The selection was made based on local community practices, the reconnaissance survey, and their availability in the study areas. The samples were collected from areas rich in plant biodiversity and natural forest resources (Rumicha, Belew, et al. 2025).

**FIGURE 2:**
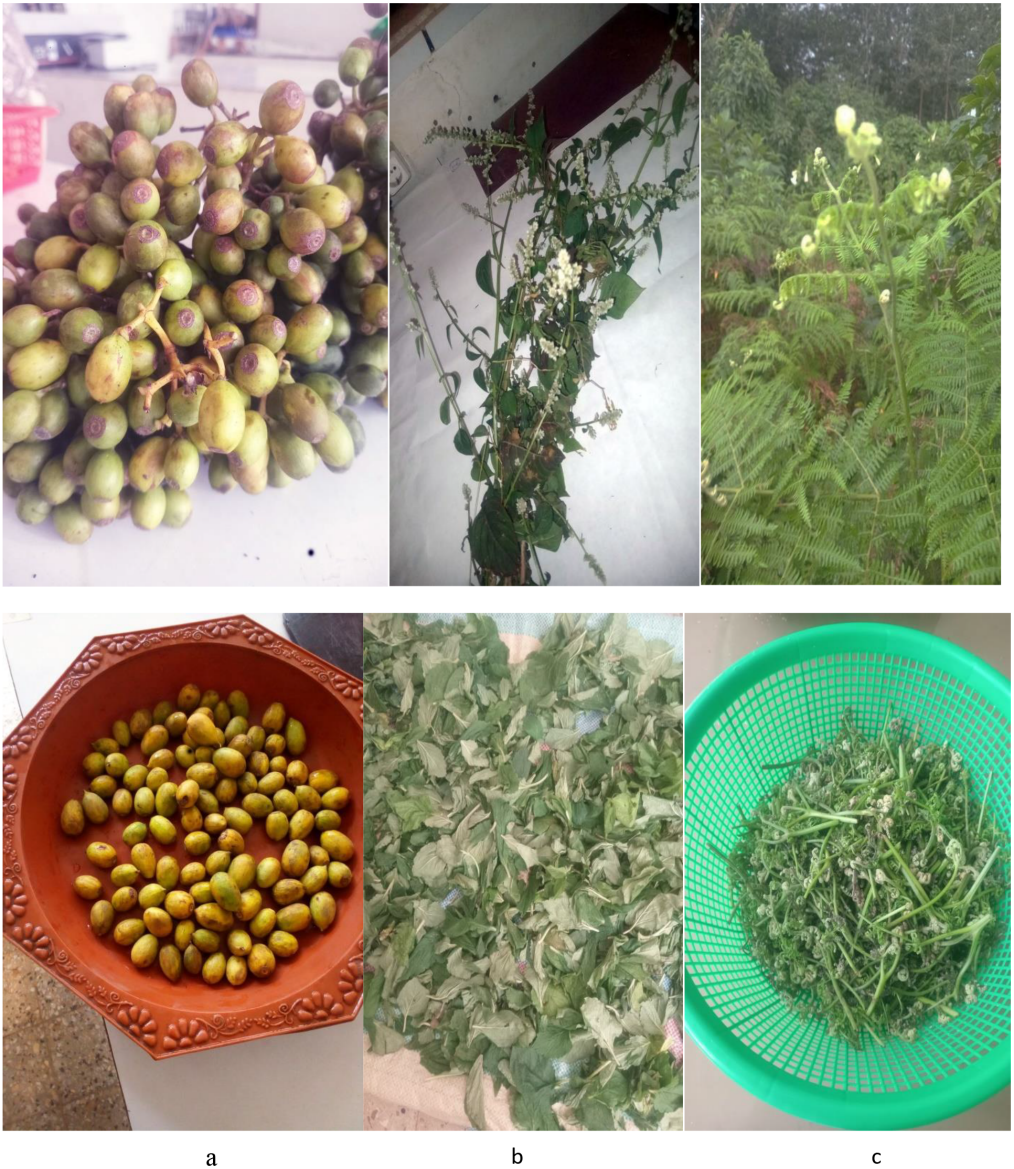
Wild edible plant species. (a) *Mussaenda arcuata* fruits, (b) *Celosia trigyna* leaves, and (c) *Pteridium aquilinum* fronds.

The first sampling site was in the Konta Zone, in the special distribution of Konta and the Seri Shewa Kebele (kebele is the lowest administrative level in Ethiopia). The kebele lies between approximately 6°49′ to 6°59′ N latitude and 36°36′ to 36°43′ E longitude. The fruits of *Mussaenda arcuata* (locally known as Mentserko) (Figure 2a) were collected from this area.

**FIGURE 3:**
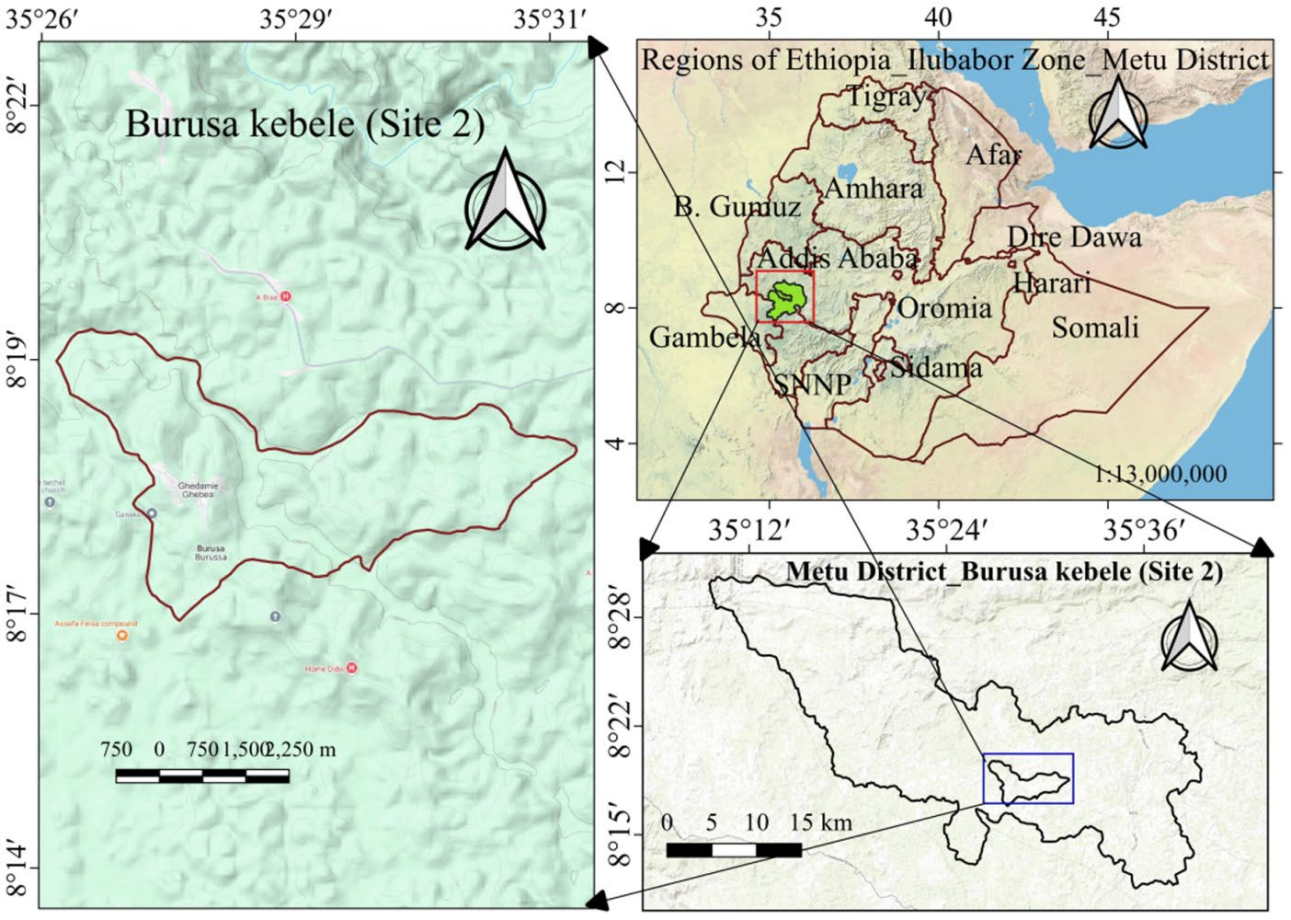
Map of the study site 2 (Burusa Kebele) to collect samples of *Celosia trigyna* leaves (Amaasillo) and *Pteridium aquilinum* (Giixoo).

The second sampling site was the Ilubabor Zone, within the Metu district, specifically in Burrusa Kebele. This site was chosen for the collection of *Celosia trigyna* leaves (Amaasillo) and *Pteridium aquilinum* (Giixoo) fronds (Figure 2b,c). The latitude range extends from 8°15′30″ N to 8°21′0″ N, while the longitude range extends from 35°27′30″ E to 35°31′30″ E (Figure 3). The region's diverse ecological landscape makes it an ideal site for research on edible wild plants, helping to better understand their distribution and availability.

### 2.2 Sample Preparation

The samples were collected and transported to the Jimma University College of Agriculture and Veterinary Medicine Post Harvest Management Laboratory with an icebox and stored in a freezer at −4°C until analysis.

### 2.3 Experimental Design and Treatments

The samples were dried at three different drying temperatures in a completely randomized design (CRD) in triplicate. The bioactive compounds of the dried wild edible plants were analyzed at each drying temperature, and the most favorable drying temperature was identified for better retention of the bioactive compounds. The wild edible plants dried at the best drying temperature identified were packed in polyethylene bags and stored at room temperature (20°C–25°C) to investigate the storage stability of bioactive compounds for 12 months CRD was also used for the storage study in triplicate, and the parameters were analyzed at 0, 2, 4, 8, and 12 months of storage.

### 2.4 Drying Treatment

The drying experiment was carried out using a convective air oven (DHG‐9203A, Shanghai, China). Fresh and healthy fruits, leaves, and fronds of the collected samples were cleaned and trimmed before sample preparation for analysis. The samples were dried in a thin layer at 60°C, 65°C, and 70°C until there was no change in weight difference between the consecutive measurements (Muliterno et al. 2017; Faustino et al. 2007; Akpinar and Bicer 2005; Ertekin and Yaldiz 2004). The *Mussaenda arcuata* fruits and *Pteridium aquilinum* fronds were cut to size using a sharp knife. Based on measurements taken with the caliper, a uniform thickness of 2 mm was maintained for thin‐layer drying models. The same is true for *Celosia trigyna* leaves, and the best‐fit mathematical model was also evaluated and identified. The samples were dried until they reached a constant weight and then ground using a laboratory mill before analysis. The samples were then analyzed for ascorbic acid, β‐carotene, total flavonoid, and total phenolic content.

### 2.5 Packaging and Storage of the Dry Samples

The dried samples were packed in double‐layer low‐density polyethylene bags to minimize air and moisture diffusion and stored at room temperature (20°C–25°C) for 12 months. Low‐density polyethylene bags were selected due to their availability, cost, and wider use in the food industries of the country. Enough samples were stored in triplicate and analyzed after 4, 8, and 12 months of storage. The initial sample before storage time was considered a zero‐time sample to compare the parameter change with storage time.

### 2.6 Data Collected

#### 2.6.1 Determination of Ascorbic Acid Content

The ascorbic acid content of the dried samples was analyzed using the titration method according to AOAC (2005). For the extraction, 10 g of dried sample powder was weighed and transferred to a 250‐mL beaker. Then, 50 mL of hot distilled water was added, and the mixture was stirred for an hour. After blending, the extract was filtered through cheesecloth and washed with 10 mL of distilled water. The filtrate was diluted to 100 mL with distilled water. A 20 mL aliquot of the sample solution was pipetted into a 250 mL conical flask, followed by the addition of 150 mL of distilled water and one milliliter of starch indicator. The solution was titrated with 0.005 M iodine solution until a persistent blue‐black color appeared, indicating the endpoint of the titration. The best drying temperature favorable to better retention of ascorbic acid was identified to indicate better nutritional retention at a specified drying temperature.

#### 2.6.2 Determination of β‐Carotene

The β‐carotene content was determined by spectrophotometry using the method described by Sadler et al. (1990). One gram of the samples was placed in a 125‐mL flask; 100 mL of hexane–acetone–ethanol (50:25:25) and an extraction solvent containing 0.1% butylated hydroxytoluene (BHT) were added to the flask. The suspension was wrapped in aluminum foil and agitated for 10 min on a wrist action homogenizer (PLTYRON2500E, Switzerland). The solution was then mixed with one gram of CaCl_2_·2H_2_O and gently shaken for 30 min with a mechanical shaker. After adding 15 mL of distilled H_2_O, the solution was shaken again for 15 min. The organic phase containing β‐carotene was separated from the water phase using a separation funnel and filtered using Whatman number 1 filter paper.

Sample handling, homogenization, and extraction were performed under low light and at 4°C to minimize photoisomerization and oxidation of carotenoids. Each sample was analyzed three times, and an average value was calculated. The β‐carotene content in the sample and β‐carotene standard (Sigma Aldrich) was estimated from absorbance read at 450 nm using spectrophotometry (V‐630; JASCO, Japan). The standard solution of stock β‐carotene (Sigma Aldrich from the USA) was made by accurately weighing 0.01 g of β‐carotene in the same solvent used to extract samples and bringing the volume to 100 mL. After the standard curve analysis, the β‐carotene content was calculated using a regression equation and expressed as mg per 100 g of edible portion.

#### 2.6.3 Determination of Total Flavonoid Contents (TFC)

The total flavonoid content (TFC) of dried flour extracts was estimated using the colorimetric method (Tanvir et al. 2017). Briefly, one milliliter of extract with a concentration of 0.1 g/mL was mixed with 0.3 mL of 5% sodium nitrite. After 5 min, 0.3 mL of 10% aluminum chloride was added, followed by 2 mL of one molar sodium hydroxide after 6 min of incubation, and the immediate addition of 2.4 mL of distilled water to produce a total volume of 10 mL. The color intensity of the flavonoid–aluminum complex was measured at 510 nm using a spectrophotometer (V‐630; JASCO, Japan). Finally, TFC was determined against the (+)‐catechin standard (Sigma Aldrich) (*R* ^2^ = 0.988) as (+)‐catechin equivalent (CE) in a concentration range of 1.00–100.00 μg/mL, and the result was expressed as milligrams of CE/100 g of sample flour.

#### 2.6.4 Determination of Total Phenolic Compounds (TPC)

The total phenolic content (TPC) of the flour extracts was determined according to the Folin–Ciocalteu method adopted by Tanvir et al. (2017). Consequently, 0.5 mL of the extract was mixed with 1.6 mL of 7.5% NaCO_3_ solution, followed by the addition of two milliliters of 10‐fold diluted Folin–Ciocalteu reagent reaction mixture to be incubated for one hour in the dark. The intensity of the blue complex was measured spectrophotometrically at a wavelength of 765 nm using a spectrophotometer (V‐630; JASCO, Japan). Finally, the TPC present in the samples was determined using the gallic acid standard (GAE) (SPECTRUM CHEMICAL, China) (*R* ^2^ = 0.999) as gallic acid equivalent (GAE) in concentration ranges of 12.50–100.00 mg/L and expressed as milligram of GAE/100 g of the flour sample.

### 2.7 Moisture Ratio (MR)

Moisture ratio (MR) will be calculated according to the method described by Menges and Ertekin (2006).

(1) $$\mathrm{MR}=\frac{M-M_{e}}{M_{i}-M_{e}}$$

where, *M* = Moisture content (db) at time *t*; *M*_*e*_ = equilibrium moisture content (db); *M*_*i*_ = Initial moisture content (db) at time *t* = 0.

### 2.8 Effective Moisture Diffusivity (*D*_eff_)

The drying behavior of agricultural products during the falling rate period can be described using Fick's second law of diffusion, which models moisture movement as a mass transfer process. The general form of the diffusion equation is expressed as (Doymaz 2010).

(2) $$\frac{\partial M}{\partial t}=D_{\mathrm{eff}}\;\nabla^{2}\;M$$

where, *M* = the moisture content, *t* = time (s), *D*_eff_ = the effective moisture diffusivity (m^2^/s), ∇^2^ *M* = the Laplacian representing the spatial distribution of moisture.

For slab geometry, assuming a uniform initial moisture distribution, negligible external resistance to mass transfer, constant diffusivity and negligible shrinkage, Crank and Gupta (1975) derived a solution to Equation (2) as follows:

(3) $$\mathrm{MR}=\frac{8}{\pi^{2}}\sum_{n=0}^{\infty }\frac{1}{{\left(2n+1\right)}^{2}}\exp\frac{{\left(2n+1\right)}^{2}\pi D_{\mathrm{eff}}t}{4L^{2}}$$

where MR = moisture ratio, *L* = half thickness of the sample (m), *n* = a positive integer.

For longer drying times, the higher‐order terms in the series become negligible, and Equation (3) can be approximated in logarithmic form.

(4) $$\ln\mathrm{MR}=\ln\frac{8}{\pi^{2}}-\left(\frac{\pi^{2}D_{\mathrm{eff}}t}{4L^{2}}\right)$$

This linearized form suggests that ln (MR) versus time *t* yields a straight line. The slope of this line, *K*, is related to the effective diffusivity of moisture as follows:

(5) $$K=\frac{\pi^{2}D_{\mathrm{eff}}}{4L^{2}}$$

From this slope, the value of *D*_eff_ can be determined using the following.

(6) $$D_{\mathrm{eff}}=\frac{4L^{2}K}{\pi^{2}}$$

where *D*_eff_ = the effective moisture diffusivity (m^2^/s), *L* = the thickness of the cut sample (m), *K* = Slope of the graph of ln (MR) again to observe the drying time.

### 2.9 Evaluation of Fitting Mathematical Models for Drying

Modeling the drying condition at a specified temperature is vital for the production of high‐quality and shelf‐stable products by controlling and optimizing process parameters (Clemente et al. 2011). To achieve this, data on drying kinetics and the evaluation of their mathematical models are essential in designing, simulating and optimizing the drying temperature. Seven relevant mathematical models for drying indicated in Table 1 recommended for the drying of fruits and vegetables, were evaluated for thin‐layer drying.

**TABLE 1:** Selected thin‐layer mathematical models to predict drying time of wild edible plants.

Model name	Model	References
Newton/Lewis	$\mathrm{MR}=\frac{\left(M-M_{e}\right)}{\left(M_{0}-M_{e}\right)}=\exp^{\left(-k_{0^{t}}\right)}$	Sarsavadia et al. (1999), Ayensu (1997), Liu and Bakker‐Arkema (1997), Nellist (1987), Tiris et al. (1994), Hummeida and El‐Sheikh (1989)
Pages/Modified Lewis	$\mathrm{MR}=\frac{\left(M_{t}-M_{e}\right)}{\left(M_{i}-M_{e}\right)}=\exp^{\left(-kt^{n}\right)}$	Agrawal and Singh (1977), Bruce (1985), Chhinnan (1984)
Henderson and Pabis/Single term	$\mathrm{MR}=\frac{M_{t}-M_{e}}{M_{i}-M_{e}}=A_{0\;\exp^{\left(-k_{0}t\right)}}$	Yagcıoglu (1999), Bengston et al. (1998), Pal and Chakraverty (1997), Rahman et al. (1997)
Logarithmic	$\mathrm{MR}=\frac{\left(M_{t}-M_{e}\right)}{\left(M_{i}-M_{e}\right)}=A_{0\;\exp^{\left(-k_{0}t\right)}}+C$	Ertekin and Yaldiz (2001), Yagcıoglu (1999)
Two‐term	$\mathrm{MR}=\frac{\left(M_{t}-M_{e}\right)}{\left(M_{i}-M_{e}\right)}=A_{0\;\exp^{\left(-k_{0}t\right)}}+A_{1\;\exp^{\left(-k_{1}t\right)}}$	Rahman et al. (1997), Madamba et al. (1996), Verma et al. (1985), Henderson (1974)
Two‐term exponential	$\mathrm{MR}=\frac{\left(M_{t}-M_{e}\right)}{\left(M_{i}-M_{e}\right)}=A_{0\;\exp^{\left(-k_{0}t\right)}}+\mathrm{bt}$	Ertekin and Yaldiz (2001), Yaldiz et al. (2001), Eldeen et al. (1980)
Diffusion approximation	$\mathrm{MR}=\frac{\left(M_{t}-M_{e}\right)}{\left(M_{i}-M_{e}\right)}=a\;\exp^{\left(-\mathrm{kt}\right)}+\left(1-a\right)\;\exp^{\left(-\mathrm{kbt}\right)}$	Ertekin and Yaldiz (2001)

The principal criterion for choosing the best model was the coefficient of determination (*R* ^2^). A higher *R* ^2^ value closer to one represented the best fit of the model (Gunhan et al. 2005). Minimum value chi‐square (χ^2^), root mean square error (RMSE), the mean bias error (MBE) and the mean relative percentage error (P%) between the predicted and observed values were described for the difference in the moisture ratio of dried samples (Goyal et al. 2008). The equations with the highest *R* ^2^ and lowest MBE, RMSE, P% and *X* ^2^ were indications of the better estimate of the drying curves, and the statistical values were calculated as follows:

(7) $$\mathrm{MR}=\frac{M-M_{e}}{M_{i}-M_{e}}$$

(8) $$R^{2}=1-\frac{\sum_{i=1}^{n}{\left(\mathrm{MR}\exp_{i}-\mathrm{MR}{\mathrm{pre}}_{i}\right)}^{2}}{\sum_{i=1}^{n}{\left(\text{MRexp mean}-\mathrm{MR}{\text{pred}}_{i}\right)}^{2}}$$

(9) $$x^{2}=\frac{\sum_{i=1}^{n}{\left(\mathrm{MR}\exp_{i}-\mathrm{MR}{\mathrm{pre}}_{i}\right)}^{2}}{N-n}$$

(10) $$\text{RMSE}={\left[\frac{1}{N}\sum_{i=1}^{n}{\left(\mathrm{MR}{\mathrm{pre}}_{i}-\mathrm{MR}\exp\text{mean}\right)}^{2}\right]}^{\frac{1}{2}}$$

(11) $$\mathrm{MBE}=\frac{1}{N}\sum_{i=1}^{n}{\left(\mathrm{MR}{\mathrm{pre}}_{i}-\mathrm{MR}\exp_{i}\right)}^{2}$$

(12) $$P\left(\%\right)=\frac{100}{N}\sum \frac{/{\mathrm{MR}}_{\exp}-{\mathrm{MR}}_{\text{pred}}/}{{\mathrm{MR}}_{\exp}}$$

where *M* = Moisture content (db) at time *t*, *M*_*e*_ = equilibrium moisture content (db), *M*_*i*_ = Initial moisture content (db) at time *t* = 0, *MRexp*_*i*_ = be the first experimental moisture ratio, *MRpre*_*i*_ = first predicted moisture ratio, *N* = number of observations, *n* = number of constants, *MRexp* mean = is the mean value of the experimental moisture ratio.

### 2.10 Determination of the Best Storage Duration (Half‐Life Time)

After identifying the most favorable drying temperature for better retention of bioactive compounds, dried samples from the identified drying temperature were packed in polyethylene bags and stored at room temperature (22°C ± 2.5°C) for 12 months. The samples were analyzed at the start (zero time) and at the end of 4, 8 and 12 months to determine the storage stability.

#### 2.10.1 Determination of Half‐Life Times (*t*_1/2_)

The half‐life time (*t*_1/2_) of a reaction refers to the time required for the concentration of a compound to decrease to half of its initial value. This concept is widely utilized in chemistry, pharmacology, and food science to model the degradation or elimination of substances over time (Snyder et al. 2011). In the context of bioactive compounds in stored plant materials or food products, the half‐life is particularly useful for estimating the time needed for these compounds to decline to 50% of their original concentration at the start of storage (zero time), thereby aiding in the assessment of shelf life and functional stability (Nicoli et al. 1999). The concept of half‐life plays a key role in determining the hold time after storage to achieve a concentration of 50% bioactive compounds compared to the initial concentration at the start of storage (zero time).

It is important to note that the half‐life time varies between different reaction types. In the following section, different types of reactions were reviewed; the derivation of their half‐life equations was explained.

To solve for the half‐life time of first‐order reactions, the rate law of a first‐order reaction is recalled (Van Holde et al. 2006).

(13) $$\left[A\right]={\left[A\right]}_{o}e^{-\mathrm{kt}}$$

It was identified as *t*_1/2_ when

(14) $$\left[A\right]=\frac{{\left[A\right]}_{0}}{2}$$

Substituting Equation (1)

(15) $$\frac{{\left[A\right]}_{o}}{2}={\left[A\right]}_{o}e^{-\mathrm{kt}\frac{1}{2}}$$

(16) $$\frac{1}{2}=e^{-\mathrm{kt}\frac{1}{2}}$$

(17) $$t_{\frac{1}{2}}=\frac{\ln2}{k}$$

(18) $$t_{\frac{1}{2}}=\frac{0.693}{k}$$

### 2.11 Data Analysis

The evaluation of mathematical models was performed using nonlinear regression analysis with the XLSTAT software package. Experimental data, collected in triplicate, were subjected to analysis of variance (ANOVA) using Minitab 17 statistical software. Before analysis, the assumptions of normality and homogeneity of variances were assessed to validate the use of parametric tests. The differences between treatment means were further examined using Tukey's post hoc test at a significance level of α = 0.05. The results were expressed as means and standard errors of the mean and presented in tabular format.

## 3 Results and Discussion

### 3.1 Effect of Drying Temperature on Bioactive Compounds

#### 3.1.1 Ascorbic Acid Content

Ascorbic acid is a water‐soluble vitamin widely present in various fruits and vegetables. It is a crucial nutrient that plays an essential role in numerous metabolic processes within the human body. It supports the proper function of key biosynthetic enzymes and acts as a cofactor in many enzymatic reactions (Hacısevki 2009). One of its most significant benefits is its antioxidant capacity, which enables it to counteract highly reactive radicals in the body (Akbari 2016).

Ascorbic acid, commonly used as an indicator of nutrient quality in fruits and vegetable food products, is highly sensitive to degradation during food processing and storage. This instability is due to its susceptibility to factors such as temperature fluctuations. In aqueous solutions, ascorbic acid is particularly unstable, as it degrades easily when exposed to normal conditions, such as heat (Santos and Silva 2008).

The present study demonstrated that drying temperature had a significant effect on the retention of ascorbic acid in the analyzed wild edible plants. *Mussaenda arcuata* retained approximately 72.9% of its initial ascorbic acid content when dried at 60°C, which declined dramatically to 41.1% at 70°C. Similarly, *Celosia trigyna* preserved 93.4% at 60°C, but only 37.4% at 70°C. *Pteridium aquilinum* showed a retention of 86.1% at 60°C, which dropped to 52.4% at 70°C (Table 2). The progressive loss of ascorbic acid with increasing temperature is attributed to its oxidation to dehydroascorbic acid, which further undergoes irreversible hydrolysis to form diketogulonic acid and other biologically inactive compounds (Lee and Kader 2000).

**TABLE 2:** Bioactive compound content of dried WEPs at different temperatures.

WEPs	Drying temperature (°C)	Bioactive compound contents			
		Ascorbic acid (mg/100)	β‐Carotene (mg/100 g)	Total flavonoid content (TFC) (mg CE/100 g)	Total phenolic contents (TPC) (mg GAE/100 g)
*Mussaenda arcuata*	Fresh	132.99^a^ ± 1.02	42.25^a^ ± 0.19	113.52^a^ ± 0.92	468.79^a^ ± 1.96
	60	96.88^b^ ± 5.09	22.77^b^ ± 0.25	101.33^b^ ± 0.41	359.27^b^ ± 1.47
	65	62.82^c^ ± 0.78	15.09^c^ ± 0.15	63.75^c^ ± 0.41	335.32^c^ ± 4.98
	70	54.61^c^ ± 0.51	9.10^d^ ± 0.38	55.04^d^ ± 0.61	278.72^d^ ± 3.21
	CV (%)	3.54	2.88	1.28	1.50
*Celosia trigyna*	Fresh	53.43^a^ ± 1.28	46.44^b^ ± 0.96	98.71^a^ ± 1.01	228.52^d^ ± 1.19
	60	49.91^a^ ± 0.78	53.55^a^ ± 0.6	95.0^a^ ± 0.45	306.48^b^ ± 1.91
	65	37.28^b^ ± 0.78	46.15^b^ ± 0.50	84.30^b^ ± 0.98	326.75^a^ ± 1.77
	70	19.97^c^ ± 1.06	15.01^c^ ± 0.83	50.77^c^ ± 1.69	268.11^c^ ± 2.59
	CV (%)	4.91	4.25	2.59	1.15
*Pteridium aquilinum*	Fresh	73.98^a^ ± 0.51	33.90^b^ ± 1.23	57.32^a^ ± 0.62	334.5^a^ ± 2.81
	60	63.71^b^ ± 0.78	42.87^a^ ± 0.53	38.01^b^ ± 1.45	252.73^b^ ± 3.19
	65	51.08^c^ ± 1.01	22.97^c^ ± 0.10	30.04^c^ ± 1.89	235.73^c^ ± 2.52
	70	38.75^d^ ± 1.02	13.65^d^ ± 0.58	20.96^d^ ± 1.55	225.25^d^ ± 1.36
	CV (%)	2.83	4.15	8.04	2.39

*Note:* All the values given are means of three independent measurements ± standard error (SE). Means not followed by the same superscript letters in each column are significantly (*p* < 0.05) different from each other.

Abbreviations: CE, Catechin Equivalent; GAE, Gallic acid equivalent.

This degradation pathway is accelerated by exposure to heat and oxygen. Additionally, ascorbic acid degradation is known to follow first‐order reaction kinetics, where the degradation rate increases exponentially with temperature (Kumar et al. 2021). When processing temperatures exceed 60°C, the structural integrity of vitamin C is compromised, leading to a significant decline in its antioxidant function. The partial retention of ascorbic acid at 60°C observed in this study suggests that moderate drying conditions can balance effective moisture removal with the preservation of heat‐sensitive nutrients. Previous studies also support the use of controlled temperature drying to retain labile compounds such as vitamin C in plant materials (Sahari et al. 2004).

Furthermore, ascorbic acid is prone to oxidative destruction not only by heat and oxygen but also by enzymatic and non‐enzymatic reactions. Although some studies attribute degradation to oxidase enzymes, during thermal drying, the primary cause of loss is non‐enzymatic oxidation (Awuchi et al. 2020; Muzolf‐Panek et al. 2017).

In general, the findings of this study identified 60°C as the most favorable drying temperature to preserve the ascorbic acid content in all plant samples. This result aligns with observations by Elgamal et al. (2023), who reported that optimal temperatures for ascorbic acid retention in fruits and vegetables generally fall within the range of 40°C–60°C. Thus, maintaining drying temperatures within this range can be considered a best practice for conserving the nutritional and biofunctional quality of wild edible plants.

#### 3.1.2 β‐Carotene Content

β‐carotene is a vital precursor to vitamin A, as its structure produces two retinol molecules upon conversion (Rodriguez‐Amaya 2001). In addition to its role, it functions as a potent antioxidant due to its conjugated double bonds, which allow it to stabilize reactive intermediates. It effectively quenches singlet oxygen and scavenges free radicals, thus protecting cellular tissues from oxidative damage such as lipid peroxidation (Pénicaud et al. 2011; Laguerre et al. 2007; Pérez‐Gálvez and Mínguez‐Mosquera 2001).

The findings revealed that β‐carotene retention during drying was species‐dependent and strongly influenced by temperature. In *Mussaenda arcuata*, *the* content decreased progressively with increasing temperature, with retention dropping to 53.9% at 60°C and dropping to 21.5% at 70°C. On the contrary, *Celosia trigyna* exhibited an unexpected increase of 115.3% at 60°C, while *Pteridium aquilinum* showed an increase of 26.4% at the same temperature. These gains suggest that moderate heat treatment may enhance the release or extractability of β‐carotene from plant tissues, likely due to heat‐induced softening of cell walls and disruption of carotenoid–protein or carotenoid–fiber complexes. Such structural changes facilitate the migration of intracellular compounds, increasing their bioavailability for extraction (Yuan et al. 2009; Rodriguez‐Amaya 1997).

However, when the temperature was elevated to 70°C, all three species showed substantial declines in β‐carotene retention, specifically 32.3% in *Celosia trigyna* and 40.3% in *Pteridium aquilinum* . This sharp decrease is attributed to the thermal degradation and oxidative breakdown of β‐carotene, which is highly sensitive to heat, light, and oxygen. At higher temperatures, the compound undergoes oxidative cleavage and isomerization from its stable all‐trans form to less stable cis‐isomers, reducing the activity of provitamin A (Van den Berg et al. 2000). Prolonged heating may also accelerate lipid peroxidation, exacerbating the loss of lipophilic antioxidants such as carotenoids (Läubli and Bruttel 1986).

These findings underscore the importance of drying at moderate temperatures not only to minimize nutrient degradation but also to improve measurable bioactive levels through improved extractability. Controlling the drying temperature is, therefore, critical to optimizing the retention and functional value of β‐carotene in wild edible plants.

In general, the drying temperature plays a critical role in the preservation of β‐carotene, which is a heat‐sensitive and oxygen‐labile carotenoid abundant in fruits, vegetables, and wild edible plants. Moreover, oxidative degradation intensifies at elevated temperatures, particularly during hot air or sun drying, breaking down the polyene chain into less active compounds such as apocarotenals (Chen and Huang 1998). Empirical evidence supports this trend; for example, Mbondo et al. (2018) reported that spinach dried at 50°C retained significantly more β‐carotene than samples dried at 70°C or 90°C, where losses reached up to 56%.

Thus, drying wild edible plants at 60°C appears optimal for β‐carotene retention, thus supporting their therapeutic efficacy and nutritional value.

#### 3.1.3 Total Flavonoid Contents (TFC)

Flavonoids are a class of polyphenolic compounds characterized by a C6–C3–C6 structure consisting of three rings (A, B, and C) and include subclasses such as flavonols, flavones, isoflavones, flavanones, flavan‐3‐ols, and anthocyanidins (Khan et al. 2020; Lin et al. 2022). They are predominantly derived from fruits, vegetables, cereals, and legumes, and are well known for their health‐promoting properties, including anti‐inflammatory, anti‐aging, antioxidant, and antidiabetic activities (Ganesan et al. 2021; Teng et al. 2021; Zhao et al. 2020).

The findings of this study demonstrated that the retention of total flavonoid content (TFC) at 60°C was notably high for the three species, with *Mussaenda arcuata* retaining 89.2%, *Celosia trigyna* achieving 96.2%, and *Pteridium aquilinum* retaining 66.3%. However, at 70°C, a pronounced reduction in TFC was observed in all species, indicating that elevated drying temperatures adversely affect the stability of flavonoids. Flavonoids, a class of polyphenolic compounds with strong antioxidant, anti‐inflammatory, and antimicrobial properties, are moderately heat sensitive and can undergo structural degradation under high thermal conditions (Zhang and Tsao 2016). The loss of flavonoids at 70°C is likely due to thermal cleavage of glycosidic bonds that link flavonoids to sugar moieties, which, when broken, can lead to less stable aglycones that are more prone to oxidation (Kähkönen et al. 1999).

The relatively high retention of flavonoids at 60°C can be attributed to two complementary mechanisms. First, moderate heat may partially inactivate endogenous oxidative enzymes such as polyphenol oxidase (PPO) and peroxidase (POD), which are major contributors to postharvest flavonoid degradation. These enzymes catalyze the oxidation of flavonoid hydroxyl groups, forming quinones that subsequently polymerize into non‐functional compounds. Their thermal inactivation at 60°C helps preserve the integrity of native flavonoids (Nicoli et al. 1999). Second, the relatively low drying temperature imposes minimal thermal stress on the flavonoid molecules. Many glycosylated flavonoids, such as quercetin glucosides and kaempferol derivatives, exhibit a degree of thermal stability at moderate temperatures and can retain their antioxidant capacity without significant degradation or isomerization (Patras et al. 2010). On the contrary, excessive heat (typically > 100°C) has been shown to cause structural breakdown or conversion to less bioactive forms (Turkmen et al. 2005).

Drying at 60°C, therefore, appears to strike a favorable balance sufficient to reduce moisture content and inhibit microbial and enzymatic spoilage, while still gentle enough to avoid thermal degradation of sensitive phytochemicals. Similar results have been reported in leafy vegetables and herbal materials, where drying at 50°C–60°C retained high levels of flavonoids and preserved antioxidant activity (Rossi et al. 2003; Dewanto et al. 2002). On the contrary, elevated temperatures have consistently been associated with higher losses due to oxidation, thermal breakdown, and enzyme‐mediated degradation (Chan et al. 2007; Wojdyło et al. 2009).

The lower loss of total flavonoid contents observed in selected WEPs at drying temperatures between 60°C and 70°C might be due to oxidation reactions of hydroxyl groups that produced more polyphenols or monophenols to di‐or triphenols. Loss of total flavonoid content can also occur during typical macromolecule degradation during heat treatment, depending on drying temperature and time (Mohd Zainol et al. 2009). According to Schieber et al. (2001), the loss of phytochemicals such as flavonoids during heat treatment might be due to the harsh drying conditions, particularly the temperature and duration used.

Therefore, drying wild edible plants at 60°C can be considered an optimal condition to maximize flavonoid retention. This temperature not only minimizes thermal degradation but also may enhance extraction capacity and preserve the functional and therapeutic value of these bioactive compounds.

#### 3.1.4 Total Phenolic Contents (TPC)

Phenolic compounds widely distributed in medicinal plants, pharmaceuticals, spices, vegetables, fruits, wild edible plants, grains, and other seeds are an important group of natural antioxidants with possible beneficial effects on human health (Pandey and Rizvi 2009). Polyphenols are widely considered very unstable and highly susceptible to degradation (Bąkowska et al. 2003). The stability of polyphenols under different conditions is an important aspect that has to be taken into account to ensure that phenolic compounds have the desired properties and maintain their activity and structure during the different stages of processing, which can involve high temperatures, light, oxygen, solvents, the presence of enzymes, proteins, metallic ions, or association with other food constituents (Casta Eda‐Ovando et al. 2009).

The study revealed differential retention and enhancement of phenolic compounds in wild edible plants subjected to thermal treatment at 60°C. Specifically, *Mussaenda arcuata* retained 76.6% of its original phenolic content, while *Pteridium aquilinum* retained 75.5%. On the contrary, *Celosia trigyna* exhibited a remarkable 34.1% increase in phenolic concentration compared to its fresh state. The observed variation in the initial temperature for phenolic degradation may be attributed to differences in the matrix composition and structural organization of plant tissues among species. *Leaves of Celosia* could have a more complex or tightly bound phenolic–protein or phenolic–fiber matrix, which could confer greater thermal stability to phenolic compounds (Howard et al. 1999; Nicoli et al. 1999). Additionally, variations in phenolic compound types (e.g., flavonoids vs. phenolic acids), their degree of polymerization, or interactions with other cellular constituents such as minerals or antioxidants can influence thermal resilience (Dai and Mumper 2010). The relatively lower degradation temperatures in *Mussaenda arcuata* fruits and *Pteridium aquilinum* fronds suggest a more labile phenolic profile or a less protective cellular environment, leading to earlier thermal breakdown under heating (Li et al. 2020; Michalska et al. 2018).

The increase in phenolic contents at 65°C in *Celosia trigyna* might also be due to the thermal disruption of plant cellular structures, which facilitates the release of bound phenolic compounds that are otherwise inaccessible in the intact plant matrix. Thermal processing at moderate temperatures can promote the hydrolysis of phenolic glycosides and the breakdown of complex components of the cell wall, such as cellulose, hemicellulose, and lignin, thus liberating phenolics previously conjugated to these macromolecules (Yao and Ren 2011; Naczk and Shahidi 2006). The enhanced extraction capacity of phenolics during moderate heat treatment has been documented in several plant species. For example, Mehmood and Zeb (2023) reported that mild thermal processing increased the free phenolic content of spinach leaves due to cell wall degradation and subsequent release of bound phenolics. Similarly, Juániz et al. 2016 observed that heating facilitated the liberation of phenolic acids bound to dietary fibers, leading to an increase in measurable phenolic compounds.

However, phenolic compounds are also susceptible to thermal degradation at elevated temperatures or prolonged heating durations. High heat can induce oxidative degradation, polymerization, and condensation reactions of phenolics, resulting in a decrease in their concentration and antioxidant capacity (Xu and Chang 2008). For example, catechins and flavonoids can undergo oxidative polymerization, reducing their bioavailability and functional efficacy (Manach et al. 2004). Therefore, the thermal stability of phenolics is highly dependent on temperature, time, and plant matrix composition.

In general, these findings underscore the complex interplay between thermal processing and phenolic bioavailability. Moderate heat may enhance phenolic extractability and biofunctionality by breaking down plant cell walls and releasing bound compounds, while excessive heat risks degrading these valuable phytochemicals. Therefore, optimizing the thermal treatment parameters is crucial to maximize the nutritional and therapeutic potential of wild edible plants.

### 3.2 Drying Kinetics and Activation Energy of Dried Wild Edible Plants

As stated in Table 3, the drying behavior of wild edible plants (WEP) such as *Mussaenda arcuata*, *Celosia trigyna* , and *Pteridium aquilinum* demonstrates clear temperature dependence in terms of both the drying rate constant (k) and the effective moisture diffusivity. Across the three species, both the drying rate constant (k) and the moisture diffusion coefficient (*D*_eff_) increased with increasing drying temperature from 60°C to 70°C, aligning with classical drying theory that suggests higher thermal energy enhances the mobility of the water molecule and reduces resistance to internal mass transfer resistance (Doymaz 2004; Midilli et al. 2002). For example, *Mussaenda arcuata* showed a more than 20‐fold increase in *D*_eff_ from 1.07 × 10^−8^ m^2^/s at 60°C to 2.22 × 10^−7^ m^2^/s at 70°C, highlighting its high thermal responsiveness. Similarly, its drying constant nearly doubled over the same temperature range, indicating accelerated moisture removal due to reduced water binding forces.

**TABLE 3:** Constant drying kinetics, activation energy, and diffusion coefficient of selected wild edible plants in Ethiopia.

Wild edible plant	Drying temperature	Drying constant K	Diffusion coefficient (m^2^/s)	Activation energy (J/gmol)
*Mussaenda arcuata*	60°C	0.0661	1.07E‐8	0.54
	65°C	0.0953	1.55E‐7	0.78
	70°C	0.137	2.22E‐7	1.14
*Celosia trigyna*	60°C	0.070	1.25E‐9	0.57
	65°C	0.095	1.70E‐9	0.79
	70°C	0.144	2.58E‐9	1.17
*Pteridium aquilinum*	60°C	0.075	1.22E‐7	0.61
	65°C	0.093	1.51E‐7	0.77
	70°C	0.109	1.77E‐7	0.887

Among the species studied, *Mussaenda arcuata* exhibited the highest diffusion coefficient and constant drying at all temperatures, suggesting that it has a more porous or less lignified structure that facilitates moisture movement. On the contrary, *Celosia trigyna* consistently recorded the lowest values of the diffusion coefficient values (in the range of 10^−9^ m^2^/s), which implies a denser tissue matrix or stronger water‐binding interactions, possibly due to the higher fiber or mucilage content. This suggests that *Celosia trigyna* might require longer drying times or pre‐processing methods such as enzymatic treatment or blanching to enhance drying efficiency (Krokida et al. 2001).

The activation energy (Ea), which reflects the energy barrier for moisture diffusion, further supports these findings. The activation energy increased with temperature in all species, indicating that a higher thermal input is necessary to overcome internal resistance to moisture migration. *Mussaenda arcuata* and *Celosia trigyna* showed a sharp increase in Ea from 0.54 to 1.14 J/g·mol and 0.57 to 1.17 J/g·mol, respectively, suggesting that these plants contain a substantial proportion of bound water that becomes mobilized only at elevated temperatures. In comparison, *Pteridium aquilinum* exhibited a more modest increase in Ea (from 0.61 to 0.89 J/g·mol), implying a relatively lower energy requirement for moisture removal and more stable drying behavior under varying thermal conditions (Guiné 2018).

From an application perspective, these kinetic parameters are essential for designing efficient drying protocols that minimize energy input while retaining the nutritional and functional qualities of WEPs. Higher drying rates and diffusion coefficients, as observed in *Mussaenda arcuata*, are desirable for commercial‐scale drying where throughput and energy efficiency are critical. However, species like *Celosia trigyna* , despite slower kinetics, may benefit from low‐temperature drying to preserve thermolabile bioactives, necessitating a trade‐off between drying speed and quality retention (Araya‐Farias and Ratti 2008; Akpinar et al. 2003). Overall, understanding the drying kinetics and activation energies of these underutilized edible plants not only contributes to optimizing postharvest processing but also supports their valorization as functional food ingredients in line with the goals of the sustainable food system (Guiné 2018).

### 3.3 Drying Temperature and Time of Wild Edible Plants

The drying behavior of the three studied wild edible plants, *Mussaenda arcuata*, *Celosia trigyna* , and *Pteridium aquilinum* , demonstrated a clear inverse relationship between drying temperature and drying time. As the drying temperature increased from 60°C to 70°C, the time required for complete drying decreased significantly for all species. For example, *Mussaenda arcuata* required 104 min at 60°C, which was reduced to 52 min at 70°C. A similar trend was observed for *Celosia trigyna* and *Pteridium aquilinum* , whose drying times decreased from 96 to 48 min and from 120 to 80 min, respectively (Table 4). This behavior is consistent with fundamental drying kinetics, where higher temperatures increase the rate of moisture removal by accelerating the vapor pressure gradient and improving the diffusivity of water within plant tissues.

**TABLE 4:** Drying temperature and time of selective wild edible plants.

Wild edible plant	Thickness of slice (mm)	Drying temperature (°C)	Time (minutes)
*Mussaenda arcuata*	2	60	104
	2	65	72
	2	70	52
*Celosia trigyna*	0.21	60	96
	0.21	65	66
	0.21	70	48
*Pteridium aquilinum*	2	60	120
	2	65	108
	2	70	80

Among the three plants, *Pteridium aquilinum* consistently exhibited the longest drying times at all temperatures. This extended drying period could be attributed to several factors, including higher initial moisture content, a denser tissue structure, or lower porosity, all of which can impede internal moisture migration. On the contrary, *Mussaenda arcuata* dried faster than *Pteridium aquilinum* , despite having the same slice thickness, suggesting that species‐specific anatomical and compositional factors also influence drying efficiency. Similar observations have been reported in previous studies, where plant morphology, cellular structure, and moisture distribution were found to significantly affect drying behavior and kinetics (Doymaz 2007; Mayor and Sereno 2004).

From a processing point of view, increasing the drying temperature offers advantages in reducing processing time and energy consumption. However, higher temperatures can also pose risks of thermal degradation, especially in edible wild plants valued for their nutritional and biofunctional properties, such as antioxidants, vitamins, and phytochemicals. Therefore, selecting an optimal combination of drying temperature and slice thickness is essential to achieve a balance between efficiency and retention of bioactive compounds. These findings are particularly relevant for the development of shelf‐stable wild edible plant products and their integration into functional food systems aimed at improving nutritional diversity and nutritional security.

### 3.4 Selection of the Best Mathematical Drying Models

The analysis of bioactive compounds at various drying temperatures revealed that 60°C is the most effective temperature for preserving these compounds in wild edible plants (WEP). This temperature maintained an effective equilibrium between rapid moisture extraction and the protection of delicate phytochemicals, as indicated by the observed diffusivity and activation energy values. Therefore, accurate modeling of the drying process at specified temperatures (60°C) is essential for producing high‐quality, shelf‐stable products by allowing better control and optimization of the process parameters to preserve the nutritional and functional properties of the material (Clemente et al. 2011). To achieve this, reliable data on drying kinetics and the application of appropriate mathematical models are critical for designing, simulating, and optimizing drying operations.

In this context, seven commonly used thin‐layer drying models recommended in the literature for drying fruits and vegetables were evaluated to determine their suitability to describe the drying behavior of WEPs. The best fit model for each sample was selected based on several statistical indicators, including the highest coefficient of determination (*R* ^2^) and the lowest values for the mean relative percent error (P%), the root mean square error (RMSE), the mean biased error (MBE), and the chi‐square (χ^2^). These statistical values varied depending on the type of wild edible plant studied. Selecting the most appropriate mathematical model is crucial to understanding drying mechanisms, predicting drying kinetics, optimizing process conditions to preserve product quality, improving energy efficiency, supporting the design and scaling of drying systems, determining key kinetic parameters, and enabling the standardization and comparison of results across studies.

In the fruit of *Mussaenda arcuata*, the *R* ^2^, P%, RMSE, MBE, and *X* ^2^ ranged from 0.9967 to 0.13988, 0.4660 to 5.24344, 0.2699 to 0.296155, 0.00027 to 0.07460, and 0.00033 to 0.09495, respectively (Table 5). Based on the evaluation criteria, the appropriate drying model was the Henderson and Pabis model for drying the *Celosia trigyna* leaf and *Mussaenda arcuata* fruit. The finding was consistent with the previous work by Norhadi et al. (2020) in Malaysia for oven‐dried mango at 60°C.

**TABLE 5:** Evaluation of models for WEPs dried at 60°C.

WEPs	Statistical Parameters	Mathematical models						
		Lewis	Pages model	Henderson and Pabis	Logarithmic	Two‐term	Two‐term exponential	Diffusion approximation
*Mussaenda arcuata*	*R* ^2^	0.9962	0.9298	**0.9967**	0.9967	0.9967	0.1398	0.9963
	*X* ^2^	0.00035	0.00565	**0.00033**	0.00036	0.00040	0.09495	0.00041
	RMSE	0.29077	0.26113	0.29615	0.29615	0.29615	0.26990	0.29068
	MBE	0.00032	0.00479	**0.00028**	0.00029	0.00037	0.07460	0.00032
	(P%)	0.4660	1.5449	**0.4509**	0.6553	0.4509	5.2434	0.4659
*Celosia trigyna*	*R* ^2^	0.9907	0.8045	**0.9923**	0.9862	0.9923	0.1511	0.9909
	*X* ^2^	0.00083	0.01410	**0.00079**	0.00154	0.00094	0.07601	0.00097
	RMSE	0.28769	0.24867	0.29631	0.29722	0.29629	0.26524	0.28950
	MBE	0.00077	0.01209	**0.00067**	0.00121	0.00068	0.05972	0.00076
	(P%)	0.6399	2.0767	0.6578	1.0510	0.6576	4.7533	0.63992
*Pteridium aquilinum*	*R* ^2^	0.9830	0.8999	0.9836	0.9823	**0.9877**	0.2527	0.9831
	*X* ^2^	0.00170	0.00940	0.00224	0.00229	**0.00177**	0.12126	0.00201
	RMSE	0.28512	0.26537	0.32020	0.29853	0.29999	0.26076	0.28512
	MBE	0.00138	0.00705	0.00168	0.00157	**0.00111**	0.08337	0.00138
	(P%)	0.8680	2.0507	0.9516	0.9810	**0.8127**	4.9296	0.86798

*Note:* Bold values indicate the statistically significant best fit for the selected appropriate models.

The model for the drying of the *Pteridium aquilinum* frond was a two‐term model, which is in agreement with the study conducted in Malaysia on pumpkin drying kinetics; the goodness‐of‐fit model was identified as a two‐term model (Onwude et al. 2016).

### 3.5 Storage Stability Study

#### 3.5.1 Ascorbic Acid Content During Storage

The study findings revealed that ascorbic acid retention during storage varied significantly among the three wild edible plants (WEPs) examined. *Mussaenda arcuata* retained 52.43% of its initial ascorbic acid content after 4 months of storage, which decreased sharply to 21.82% at the end of one year. Similarly, *Celosia trigyna* exhibited 57.06% retention at 4 months, decreasing to 25.83% after 12 months of storage. *Pteridium aquilinum* showed a similar trend, with 57.75% retention at 4 months, dropping to 23.73% after one year (Table 6). These results demonstrate that ascorbic acid is highly unstable over time, with all three species experiencing more than 70% degradation over the 12‐month storage period. As a water‐soluble and highly labile compound, ascorbic acid is particularly susceptible to oxidative degradation in the presence of oxygen, light, heat, and metal ions (Lee and Kader 2000). Among the WEPs studied, *Mussaenda arcuata* exhibited the most rapid loss (final retention of 21.82%), which may be attributed to a soft tissue structure or lower concentrations of endogenous protective compounds such as polyphenols and flavonoids (Dai and Mumper 2010). On the contrary, *Pteridium aquilinum* retained slightly more ascorbic acid (23.73%), possibly due to a denser, more fibrous matrix and a stronger antioxidant environment that helps mitigate oxidative damage. *Celosia trigyna* , with intermediate retention (25.83%), may have a moderately protective phytochemical profile that contributes to its relative stability.

**TABLE 6:** The contents of bioactive compounds of WEPs consumed in Ethiopia dried at 60°C.

WEPs	Storage time (months)	Bioactive compound contents							
		Ascorbic acid (mg/100 g)	(%) of retention	β‐Carotene (mg/100)	(%) of retention	Total flavonoid content (TFC) (mg CE/100 g)	(%) of retention	Total phenolic compounds (TFC) (mg GAE/100 g)	Increase/(%) of retention
*Mussaenda arcuata*	0	96.88^a^ ± 5.09		22.77^a^ ± 0.25		101.33^a^ ± 0.41		359.27^b^ ± 1.47	
	4	50.79^b^ ± 0.78	52.43	14.19^b^ ± 0.38	62.32	65.42^b^ ± 0.52	66.55	415.32^a^ ± 5.56	+15.61
	8	28.77^c^ ± 1.06	29.72	10.06^c^ ± 0.27	44.18	38.33^c^ ± 0.94	37.83	376.41^b^ ± 4.14	+4.77
	12	21.14^c^ ± 0.87	21.82	5.98^d^ ± 0.17	26.26	24.25^d^ ± 0.93	23.93	290.83^c^ ± 5.93	80.95
	Half life time (months)	4.6		6.8		5.7		—	
*Celosia trigyna*	0	49.91^a^ ± 0.78		53.55^a^ ± 0.6		95.0^a^ ± 0.45		306.48^b^ ± 1.91	
	4	28.48^b^ ± 0.78	57.06	38.36^b^ ± 0.78	71.63	54.37^b^ ± 3.76	57.23	343.65^a^ ± 2.75	+12.13
	8	17.91^c^ ± 2.69	35.88	19.84^c^ ± 1.42	37.05	42.72^c^ ± 0.49	44.97	321.80^b^ ± 5.89	+5.0
	12	12.89^d^ ± 0.51	25.83	18.41^c^ ± 0.17	34.38	34.15^c^ ± 1.93	35.95	251.65^c^ ± 4.51	82.11
	Half life time (months)	5.0		5.6		6.9		—	
*Pteridium aquilinum*	0	63.71^a^ ± 0.78		42.87^a^ ± 0.53		38.01^a^ ± 1.45		252.73^b^ ± 3.19	
	4	35.52^b^ ± 1.06	57.75	23.51^b^ ± 0.79	54.84	24.88^b^ ± 0.89	65.46	282.53^a^ ± 4.14	+11.83
	8	20.84^c^ ± 1.83	32.71	17.31^c^ ± 0.06	43.38	14.27^c^ ± 0.27	37.54	266.48^b^ ± 3.53	+5.46
	12	15.12^d^ ± 0.82	23.73	9.25^d^ ± 0.22	21.58	6.78^d^ ± 0.82	17.84	205.25^c^ ± 2.52	81.21
	Half life time (months)	5.4		6.1		5.6		—	

*Note:* All the values given are means of three independent measurements ± standard error (SE). Means not followed by the same superscript letters in each column are significantly (*p* < 0 0.05) different from each other. The total phenolic content did not decrease to 50% of its initial value during the storage period; therefore, its half‐life could not be determined. + indicates the increase in the percentage of content, − indicates the percentage of loss.

Abbreviations: CE, Catechin Equivalent; GAE, Gallic Acid Equivalent.

The overall decline in ascorbic acid can also be related to residual enzymatic activity and trapped oxygen within packaging materials, even under carefully controlled drying and storage conditions (Lee and Kader 2000; Davey et al. 2000). Although double‐layered polyethylene packaging and double sealing were used in this study, some exposure to external environmental factors is still possible. Combined with insufficient control of light, humidity, or temperature, these conditions may have accelerated the degradation process.

Differences in degradation rates between species probably reflect variations in tissue structure, initial ascorbic acid content, and the concentration of endogenous antioxidants that can help mitigate oxidative stress (Bosch et al. 2013). The fruit of *Mussaenda arcuata*, having a more delicate structure, may allow faster oxygen penetration and enzymatic activity than the tougher fronds of *Pteridium aquilinum* or the leaves of *Celosia trigyna* (Kader 2002).

In particular, the highest degradation rates across all three WEPs occurred during the first four months of storage. This may be due to early‐stage redox reactions triggered by residual oxygen, moisture, and trace metals present in plant tissues, which are known to catalyze oxidative breakdown (Andersson et al. 2015). Furthermore, studies have demonstrated that vitamin C degradation during storage is accelerated by various factors, including elevated temperatures, exposure to light, and biochemical reactions, particularly those involving enzymes such as ascorbate oxidase (Pham et al. 2019; Mahnot et al. 2019). These factors, alone or in combination, probably contributed to the observed decrease in ascorbic acid content over time.

The half‐life times for the degradation of ascorbic acid during storage of 12 months are listed in Table 6. The kinetics of ascorbic acid degradation followed a first‐order reaction. The reaction constants of ascorbic acid in dried and stored wild edible plants were determined by plotting the natural logarithm of the ascorbic acid concentration (mg/100 g dm) against the storage time. Depending on storage time, the rate of degradation of ascorbic acid increased. The half‐life time was calculated as 4.6 months for *Mussaenda arcuata fruits*, 5.0 months for *Celosia trigyna* leaves, and 5.4 months for *Pteridium aquilinum fronds*. The optimal half‐life time for better ascorbic acid retention in dried and stored wild edible plants studied was recorded as 4.6 months in *Mussaenda arcuata fruits*. High retention of ascorbic acid was recorded for the dried and stored fruits of *Mussaenda arcuata*.

#### 3.5.2 β‐Carotene During Storage

The results of the findings showed that β‐carotene retention also declined over the storage period; however, the extent of degradation varied among the three wild edible plants. *Celosia trigyna* exhibited the highest stability, retaining 71.63% of its initial β‐carotene content at 4 months and 34.38% after one year. In comparison, *Mussaenda arcuata* retained 62.32% at 4 months, which decreased to 26.26% at the end of the storage period. *Pteridium aquilinum* demonstrated the lowest retention, with 54.84% at 4 months and only 21.58% after one year (Table 6). Although β‐carotene is generally more stable than ascorbic acid, it remains susceptible to degradation through oxidative and isomerization processes. The comparatively higher retention observed in *Celosia trigyna* may be attributed to the presence of protective phytochemicals or a favorable cellular matrix that mitigates oxidative damage, as has been reported for leafy vegetables with high antioxidant potential and cell wall integrity (Rodriguez‐Amaya et al. 2008). As a highly unsaturated lipophilic compound, β‐carotene is particularly vulnerable to oxidative breakdown and structural isomerization, especially when exposed to light, heat, oxygen, and trace metal ions (Rodriguez‐Amaya 2001).

The greatest losses occurred during the first 4 to 8 months of storage, a period likely influenced by differences in plant tissue integrity and packaging effectiveness. Even with double‐layered polyethylene sealing, residual oxygen, packaging permeability, and storage temperature can accelerate oxidation reactions, leading to the formation of inactive or less bioavailable β‐carotene derivatives (Tonucci et al. 1995). Additionally, the presence of pro‐oxidant compounds and residual enzymatic activity in plant tissues may further promote degradation over time. These findings are consistent with previous studies showing that dried plant‐based foods can undergo considerable carotenoid loss unless stringent controls on oxygen, temperature, and light exposure are maintained (Nguyen and Schwartz 1998).

Importantly, even when the packaging headspace is flushed with nitrogen, dissolved residual oxygen can still drive β‐carotene degradation during long‐term storage (Vásquez‐Caicedo et al. 2007). Furthermore, the oxidative breakdown of the highly unsaturated carotenoid structure contributes further to the observed decrease in the total carotenoid and β‐carotene content (Kidmose et al. 2002).

The half‐life times for β‐carotene degradation during storage of 12 months are shown in Table 6. The kinetics of degradation of β‐carotene followed a first‐order reaction. The degradation reaction constants of β‐carotene in dried and stored wild edible plants were calculated by plotting the natural logarithm of its concentration (mg/100 g dry matter) against storage duration. Depending on storage time, the β‐carotene degradation rate increased. The half‐life time was calculated as 5.6 months for *Celosia trigyna* , 6.1 months for *Pteridium aquilinum* , and 6.8 months for *Mussaenda arcuata*. The optimal half‐life time for better retention of β‐carotene in dried and stored wild edible plants was recorded as 5.6 months and was recorded in leaves of *Celosia trigyna* .

#### 3.5.3 Total Flavonoid Content During Storage

The study findings indicated that the total flavonoid content in the examined wild edible plants declined over time, although retention was relatively higher during the initial months of storage. *Mussaenda arcuata* showed a flavonoid retention of 66.55% after 4 months, which decreased substantially to 23.93% at the end of one year. *Celosia trigyna* retained 57.23% at 4 months and 35.95% after 12 months, making it the most stable among the three species in terms of flavonoid preservation. On the contrary, *Pteridium aquilinum* exhibited a retention of 65.46% at 4 months but experienced the steepest decline, falling to just 17.84% at the end of the storage period (Table 6).

This degradation is largely attributable to oxidative and enzymatic breakdown, processes to which flavonoids are increasingly susceptible over prolonged storage, especially under suboptimal environmental conditions. Although flavonoids are generally more stable than ascorbic acid or β‐carotene, they can still undergo degradation through oxidative, enzymatic, and hydrolytic pathways when exposed to oxygen, heat, moisture, and storage time (Manach et al. 2004).

Differences in retention between species probably reflect variations in tissue structure and phytochemical composition (Li et al. 2020). *The* relatively high retention of flavonoids in *Celosia trigyna* suggests the presence of a more protective tissue matrix or a higher initial concentration of stable flavonoid compounds. On the contrary, the low retention in *Pteridium aquilinum* (17.84%) may result from rapid degradation due to enzymatic oxidation and limited intrinsic antioxidant defenses. *Mussaenda arcuata* demonstrated intermediate stability, reflecting a moderate level of protection. These species‐specific differences may also be influenced by the diversity of flavonoid subclasses present (e.g., flavonols, flavones, and anthocyanins), their glycosylation patterns, and their interactions with other phytochemicals such as phenolic acids or proteins, all of which can affect their susceptibility to degradation (Panche et al. 2016).

In addition, flavonoid losses were more pronounced during the first half of the storage period, probably driven by redox reactions catalyzed by trace metals or residual enzymatic activity within the plant matrix. Despite the use of double‐sealed polyethylene packaging, factors such as residual oxygen and temperature fluctuations can further promote degradation, as observed in prior studies on dried plant products (Wachtel‐Galor 2011). These findings underscore the need for improved storage technologies, such as oxygen scavengers or modified atmosphere packaging, to better preserve flavonoid content over time.

The half‐life times for the degradation of the total flavonoid during storage of 12 months are indicated in Table 6. The kinetics of the degradation of total flavonoids followed a first‐order reaction. The rate constants for total flavonoid degradation in dried and stored wild edible plants were determined by analyzing the relationship between storage duration and the natural logarithm of total flavonoid concentration (mg CE/100 g dry matter). Depending on storage time, the total flavonoid degradation rate increased. Half‐life time was calculated as 5.6 months for *Pteridium aquilinum* fronds, 5.7 months for *Mussaenda arcuata* fruits, and 6.9 months for *Celosia trigyna* leaves. The optimal half‐life time for better retention of total flavonoid in dried and stored study wild edible plants was recorded as 5.6 months in *Pteridium aquilinum* fronds.

#### 3.5.4 Total Phenolic Compounds During Storage

The observed trend in total phenolic compounds (TPC) in all wild edible plant (WEP) samples, marked by an initial increase during the first 8 months of storage followed by a modest decline at 12 months, can be attributed to several storage‐induced biochemical changes. The early rise in TPC is likely due to the hydrolysis of bound phenolics, where storage conditions promote the breakdown of ester and glycosidic bonds, releasing free phenolic compounds that are more readily extractable (Nicoli et al. 1999). In addition, partial depolymerization of complex high‐molecular‐weight phenolics may improve solubility and extraction efficiency (Pinelo et al. 2004). The subsequent decrease in TPC after 8 months is likely a result of oxidative degradation or polymerization of phenolic molecules into insoluble or nonextractable forms. These processes can be triggered by residual oxygen, light exposure, or enzymatic activity over time (Shahidi and Naczk 2003).

Despite the decline, final TPC retention levels of approximately 81%–82% in all species indicate a strong inherent stability of phenolic compounds in these WEPs. This is particularly notable compared to more labile nutrients, such as ascorbic acid. The high retention underscores the potential of these plants as reliable sources of dietary antioxidants even during long‐term storage. Previous studies have also shown that the phenolic content of dried wild edible plants remains relatively stable for several months, with significant degradation often occurring only after prolonged periods, typically beyond 8 months. This early stability is largely due to the drying process, which reduces the moisture content and thereby inhibits enzymatic activity (Nicoli et al. 1999; Wojdyło et al. 2007). Low water activity in dried plant tissues limits both enzymatic degradation and nonenzymatic oxidation (Vega‐Mercado et al. 2007). Furthermore, phenolic compounds may remain protected by their association with plant cell wall structures or other macromolecules, shielding them from environmental stressors such as light and oxygen (Turkmen et al. 2005). When storage conditions are carefully controlled, specifically with low humidity, minimal light exposure, and airtight packaging, phenolic degradation is further minimized (Corrêa et al. 2017).

The lack of substantial degradation before the 8‐month mark suggests the presence of a lag phase in the degradation kinetics, during which phenolics remain relatively resistant until certain oxidative or structural thresholds are reached. Similar patterns of delayed degradation have been documented in stored herbs, dried fruits, and tea leaves (Chan et al. 2009), supporting the conclusion that phenolic compounds can remain stable under optimal storage conditions over long periods.

The most favorable storage time for better bioactive compound retention for the studied wild edible plants was 4.6 months. All bioactive compounds for all the studied wild edible plants were retained with a minimum of half of their initial contents.

## 4 Conclusions

This study confirmed that fresh wild edible plants, *Mussaenda arcuata*, *Celosia trigyna* , and *Pteridium aquilinum* , are valuable sources of bioactive compounds with notable health benefits. However, the preservation of these compounds depends heavily on proper drying and storage practices. A drying temperature of 60°C was found to be optimal for maintaining key bioactive components such as ascorbic acid, beta‐carotene, total flavonoids, and total phenolics in all three species. At this temperature, a favorable balance was achieved between efficient moisture removal and minimal nutrient degradation. Drying kinetic analysis revealed that the Henderson and Pabis model best fit the drying behavior of *Mussaenda arcuata* and *Celosia trigyna* , while the two‐term model was most suitable for *Pteridium aquilinum* at the same temperature, highlighting their usefulness in process optimization. Among the species studied, *Mussaenda arcuata* showed the highest drying rates and effective diffusivity, making it more suitable for commercial‐scale drying where throughput and energy efficiency are priorities. Storage trials demonstrated good retention of ascorbic acid, a sensitive marker of nutritional stability, with a half‐life of approximately 4.6 to 6 months, suggesting the potential for extended preservation of nutritional quality. Drying time was also found to vary depending on species, temperature, and slice thickness, with thinner slices drying faster but requiring careful monitoring to prevent nutrient loss. To maintain product stability and ensure longer shelf life, the use of appropriate polyethylene packaging is recommended, as it effectively limits exposure to moisture and oxygen.

## 5 Recommendations

It is recommended to provide targeted training to local farmers and food processors on optimal drying and storage techniques to improve the quality and consistency of dried wild edible plants. The use of airtight containers and storage in cool, shaded environments should be promoted to preserve nutritional value. Encouraging the consumption of these nutrient‐rich foods among vulnerable groups, particularly children and pregnant women, can help address micronutrient and bioactive compound deficiencies in rural areas. Furthermore, supporting small‐scale food enterprises, especially women's groups and cooperatives, in the processing, packaging, and marketing of dried wild edible plants can improve rural incomes, reduce postharvest losses, and improve food and health security. Integrating traditional knowledge with scientific methods will further improve product quality and marketability, fostering sustainable and resilient food systems. Government bodies and policymakers are encouraged to support and empower communities through enabling policies, infrastructure development, and capacity‐building programs.

## Author Contributions

**Tamene Daba Rumicha:** conceptualization (equal), data curation (equal), formal analysis (equal), methodology (equal), project administration (equal), software (equal), validation (equal), writing – original draft (equal), writing – review and editing (equal). **Sirawdink Fikreyesus Forsido:** conceptualization (equal), data curation (equal), formal analysis (equal), funding acquisition (equal), investigation (equal), methodology (equal), project administration (equal), resources (equal), software (equal), supervision (equal), validation (equal), writing – original draft (equal), writing – review and editing (equal). **Yetenayet Bekele Tola:** conceptualization (equal), data curation (equal), formal analysis (equal), funding acquisition (equal), investigation (equal), methodology (equal), resources (equal), software (equal), supervision (equal), validation (equal), visualization (equal), writing – original draft (equal), writing – review and editing (equal). **Abebe Yimer:** conceptualization (equal), data curation (equal), formal analysis (equal), methodology (equal), software (equal), supervision (equal), writing – original draft (equal). **Chala G. Kuyu:** conceptualization (equal), methodology (equal), project administration (equal). **Tilahun A. Teka:** conceptualization (equal), data curation (equal), methodology (equal), supervision (equal), validation (equal). **Addisalem Hailu:** conceptualization (equal), investigation (equal), methodology (equal), supervision (equal), validation (equal), writing – original draft (equal). **Frafis Hirko:** formal analysis (equal), writing – original draft (equal).

## Conflicts of Interest

The authors declare no conflicts of interest.

## Data Availability

Data sets to support this are available upon reasonable request from the author.
